# How is Indonesia coping with its epidemic of chronic noncommunicable diseases? A systematic review with meta-analysis

**DOI:** 10.1371/journal.pone.0179186

**Published:** 2017-06-20

**Authors:** Julia Schröders, Stig Wall, Mohammad Hakimi, Fatwa Sari Tetra Dewi, Lars Weinehall, Mark Nichter, Maria Nilsson, Hari Kusnanto, Ekowati Rahajeng, Nawi Ng

**Affiliations:** 1 Unit of Epidemiology and Global Health, Department of Public Health and Clinical Medicine, Umeå University, Umeå, Sweden; 2 Centre for Reproductive Health, Faculty of Medicine, Universitas Gadjah Mada, Yogyakarta, Indonesia; 3 Department of Health Behaviour, Environment and Social Medicine, Faculty of Medicine, Universitas Gadjah Mada, Yogyakarta, Indonesia; 4 Centre for Demographic and Ageing Research, Umeå University, Umeå, Sweden; 5 School of Anthropology, College of Social and Behavioral Sciences, The University of Arizona, Tucson, United States of America; 6 Department of Family Medicine, Community Medicine and Bioethics, Faculty of Medicine, Universitas Gadjah Mada, Yogyakarta, Indonesia; 7 Center for Public Health Research and Development, National Institute of Health Research and Development (NIHRD), Ministry of Health, Jakarta, Republic of Indonesia; Western Sydney University, AUSTRALIA

## Abstract

**Background:**

Chronic noncommunicable diseases (NCDs) have emerged as a huge global health problem in low- and middle-income countries. The magnitude of the rise of NCDs is particularly visible in Southeast Asia where limited resources have been used to address this rising epidemic, as in the case of Indonesia. Robust evidence to measure growing NCD-related burdens at national and local levels and to aid national discussion on social determinants of health and intra-country inequalities is needed. The aim of this review is (i) to illustrate the burden of risk factors, morbidity, disability, and mortality related to NCDs; (ii) to identify existing policy and community interventions, including disease prevention and management strategies; and (iii) to investigate how and why an inequitable distribution of this burden can be explained in terms of the social determinants of health.

**Methods:**

Our review followed the PRISMA guidelines for identifying, screening, and checking the eligibility and quality of relevant literature. We systematically searched electronic databases and gray literature for English- and Indonesian-language studies published between Jan 1, 2000 and October 1, 2015. We synthesized included studies in the form of a narrative synthesis and where possible meta-analyzed their data.

**Results:**

On the basis of deductive qualitative content analysis, 130 included citations were grouped into seven topic areas: risk factors; morbidity; disability; mortality; disease management; interventions and prevention; and social determinants of health. A quantitative synthesis meta-analyzed a subset of studies related to the risk factors smoking, obesity, and hypertension.

**Conclusions:**

Our findings echo the urgent need to expand routine risk factor surveillance and outcome monitoring and to integrate these into one national health information system. There is a stringent necessity to reorient and enhance health system responses to offer effective, realistic, and affordable ways to prevent and control NCDs through cost-effective interventions and a more structured approach to the delivery of high-quality primary care and equitable prevention and treatment strategies. Research on social determinants of health and policy-relevant research need to be expanded and strengthened to the extent that a reduction of the total NCD burden and inequalities therein should be treated as related and mutually reinforcing priorities.

## Introduction

The eight Millennium Development Goals (MDGs) have for a long time governed the global public health agenda by determining funding priorities and steering the actions of ministries, healthcare institutions, and international non-governmental organizations [1]. Regrettably, even though chronic noncommunicable diseases (NCDs) were already the world’s leading cause of death and disability in 1990 (the health-related baseline for the MDGs) [2], these were not specifically recognized or addressed in the MDG agenda. Neglecting the grave threat posed by NCDs to human health and countries’ economic development in the MDG agenda has been described as a huge failure of the international development community [3, 4]. In retrospect, there is evidence that especially in low- and middle-income countries (LMICs), the rising burden of NCDs was a reason for the unequal progress in achieving the health-related goals, including maternal and child health improvements, and the reversal of gains already made on other MDGs, such as the eradication of extreme poverty and hunger [5, 6].

Since then, the importance of developing robust evidence of the increasing global burden of NCDs and helping transform commitments into action has been continuously emphasized since the dissemination of the “Global Strategy for the Prevention and Control of Noncommunicable Diseases” adopted by the 53^rd^ World Health Assembly in 2000 [7]. Further, equitable approaches to the prevention and control of NCDs, as most prominently exemplified by the “25 by 25 Goal”, play a central role in the UN-led discussion about the post-2015 sustainable development agenda [8, 9]. The acuteness of this issue is also mirrored in the 2014 update of the WHO Global Status Report on Noncommunicable Diseases [10] and the 2015 Global Burden of Disease update [2], which both demonstrate the global burden of epidemiological transitions towards NCDs.

These data reveal a growing and disproportionate impact of the NCD epidemic in particular on LMICs in which more than three quarters of cardiovascular- and diabetes-related deaths, nearly 90% of deaths from chronic respiratory diseases, and more than two thirds of all deaths from cancer occur [10]. The magnitude of the rise of NCDs is particularly visible in Southeast Asia where still only limited resources have been used to address the rising NCD epidemic and health inequalities [11]. Similar to other LMICs, in Indonesia, social determinants of health and intra-country inequalities in health have not received explicit attention in national discussions and this creates difficulties in formulating and implementing actions that reduce health disparities and help to lessen the total burden that NCDs inflict on human suffering and the socioeconomic structure of the country.

Currently, a comprehensive and critical review of NCD-related evidence with a focus on health inequalities does not exist for Indonesia. In light of this, a systematic review of relevant literature was undertaken with the aims of: (i) illustrating the burden of risk factors, morbidity, disability, and mortality related to NCDs; (ii) identifying existing policy and community interventions, including disease prevention and management strategies; and (iii) investigating how and why an inequitable distribution of this burden can be explained in terms of the social determinants of health.

## Methods

### Data sources

We retrieved citations through a systematic review of relevant peer-reviewed research articles and non-peer-reviewed gray literature reports. In the absence of any “gold standard” for rigorous systematic gray literature search methods, we followed and combined several strategies to identify relevant gray reports for our systematic review. The gray literature was retrieved through (i) manual targeted web-based searches of homepages of major national Indonesian governmental institutions (i.e. NIHRD, the National Institute of Health Research and Development within the Ministry of Health; BAPPENAS, the Ministry of National Development Planning, Statistics Indonesia) and international nongovernmental organizations (e.g. the World Health Organization, the World Bank); (ii) pursuing references of references via “snowballing” from the reference lists and bibliographies of the reports identified through the first strategy and the peer-reviewed articles identified in PubMed; (iii) personal knowledge using the expertise of the authors. In our review, we followed largely the Prague definition of gray literature, which defines it as “manifold document types produced on all levels of government, academics, business and industry in print and electronic formats that are protected by intellectual property rights, of sufficient quality to be collected and preserved by library holdings or institutional repositories, but not controlled by commercial publishers i.e., where publishing is not the primary activity of the producing body [12]. To account for the large heterogeneity of gray literature in terms of outlet control and source expertise, we used only so-called 1^st^ tier gray literature, which demands significant retrievability and credibility [13]. We considered the following instantiations: books and book chapters, dissertations, publications from governmental agencies, policy documents, think tank publications, reports from NGOs and consulting firms, committee reports, reports from funding agencies, program evaluation reports, working and discussion papers, reports on websites.

We supplemented the gray literature searches with electronic database searches performed in PubMed (www.ncbi.nlm.nih.gov/pubmed) and in the electronic journal “Buletin Penelitian Sistem Kesehatan” (Bulletin of Health System Research; http://ejournal.litbang.depkes.go.id), which is an Indonesian open-access journal published by the NIHRD since 1997.

We ran 17 separate searches in the PubMed database using the following search terms to identify English- and Indonesian-language peer-reviewed articles published between 01/01/2000 and 01/10/2015: “chronic disease” OR “noncommunicable disease”, “cancer”, “diabetes”, “cardiovascular disease” OR “stroke” OR “myocardial infarction”, “chronic obstructive pulmonary disease” OR “lung disease”, “high blood pressure” OR “hypertension”, “overweight” OR “obesity”, “dyslipidemia”, “cholesterol”, “smoking” OR “tobacco”, “alcohol”, “exercise” OR “physical activity”, “food habits” OR “unhealthy diet”, “disability”, “activities of daily living” OR “ADL”, “functional health”, “multimorbidity” OR “comorbidity”, AND “Indonesia”. We applied both keyword searches and MeSH term (Medical Subject Headings) searches and used the Boolean operators OR and AND to combine search terms. Corresponding translations of the above terms into the Indonesian language were used to search for relevant Indonesian-language articles in the Bulletin of Health System Research. The journal provides English translations of the titles and abstracts of its publications and with five of the authors being Indonesian native speakers, the team had the necessary language proficiency to critically appraise the non-English full-texts.

### Study selection

This systematic review included articles and gray reports that examined the differential effects of social determinants on mortality, morbidity, or disability stemming from one or several of the WHO’s priority NCDs, i.e. cancer, cardiovascular diseases, chronic obstructive respiratory diseases, and diabetes as well as their common modifiable behavioral risk factors (e.g. tobacco smoking, harmful use of alcohol, unhealthy dieting, and physical inactivity) and biological risk factors (e.g. hypertension, overweight/obesity, or a combination of risk factors like the cardiometabolic syndrome). We included outcomes that were self-reported, self-reported doctor diagnosed, or measured within the study. We also considered articles and reports focusing on policy or community interventions, including disease prevention and management strategies. We chose to use the commencing date January 1, 2000 because the literature and data availability on NCDs in Indonesia has been on the rise since the early 2000s.

In order to identify relevant citations for inclusion, two levels of screening were used: First, titles, abstracts, and keywords were screened according to the eligibility criteria mentioned in Table 1. Fulfilling only one exclusion criterion was sufficient to exclude an article or report from this review. In the second level, full text articles or reports meeting the first screening level were obtained and evaluated. The initial screening, data extraction, and quality assessment were done independently by one reviewer (JS) and checked by a second reviewer (NN). Disagreements were settled by consensus with the other co-authors. The reference management software EndNote Version X6 (Thomson Reuters, NY, USA) was used to import and manage all literature for this review.

**Table 1:** Exclusion criteria.

**Formalities:**	- Published before 01/01/2000 - Written in a language other than English or Indonesian - Studies with serious ethical issues - Studies/data published in more than one paper (duplicates) (only for the meta-analysis) - Studies that failed the critical appraisal - Gray literature with moderate or low retrievability and credibility
**Study Population:**	- Study setting does not include Indonesia - Study was conducted among populations of Indonesian origin residing outside of Indonesia - Study employed no representative population sample - For studies that include both pediatric and adult populations and where it is not possible to extract data for adults separately
**Study Design:**	- Purely clinical or experimental study design; clinical or treatment guidelines; case reports - Economic evaluation studies - Synthesis research papers such as systematic reviews and meta-analyses - Methodology papers (validation studies, mathematical model testing, etc.) - Editorials, commentaries, opinion pieces, or letters
**Outcome:**	- Oral health - Mental health - Pediatric NCDs (e.g. juvenile type-1 diabetes mellitus, childhood leukemia, etc.) - Gestational conditions (e.g. diabetes, hypertension, etc.) - Developmental disabilities, mental disabilities, accident-related disabilities
**Relatedness:**	- Analysis does not address any differential effects regarding one or more social determinants of health, health inequalities or inequities, or disadvantaged or vulnerable population.

**Fig 1:**
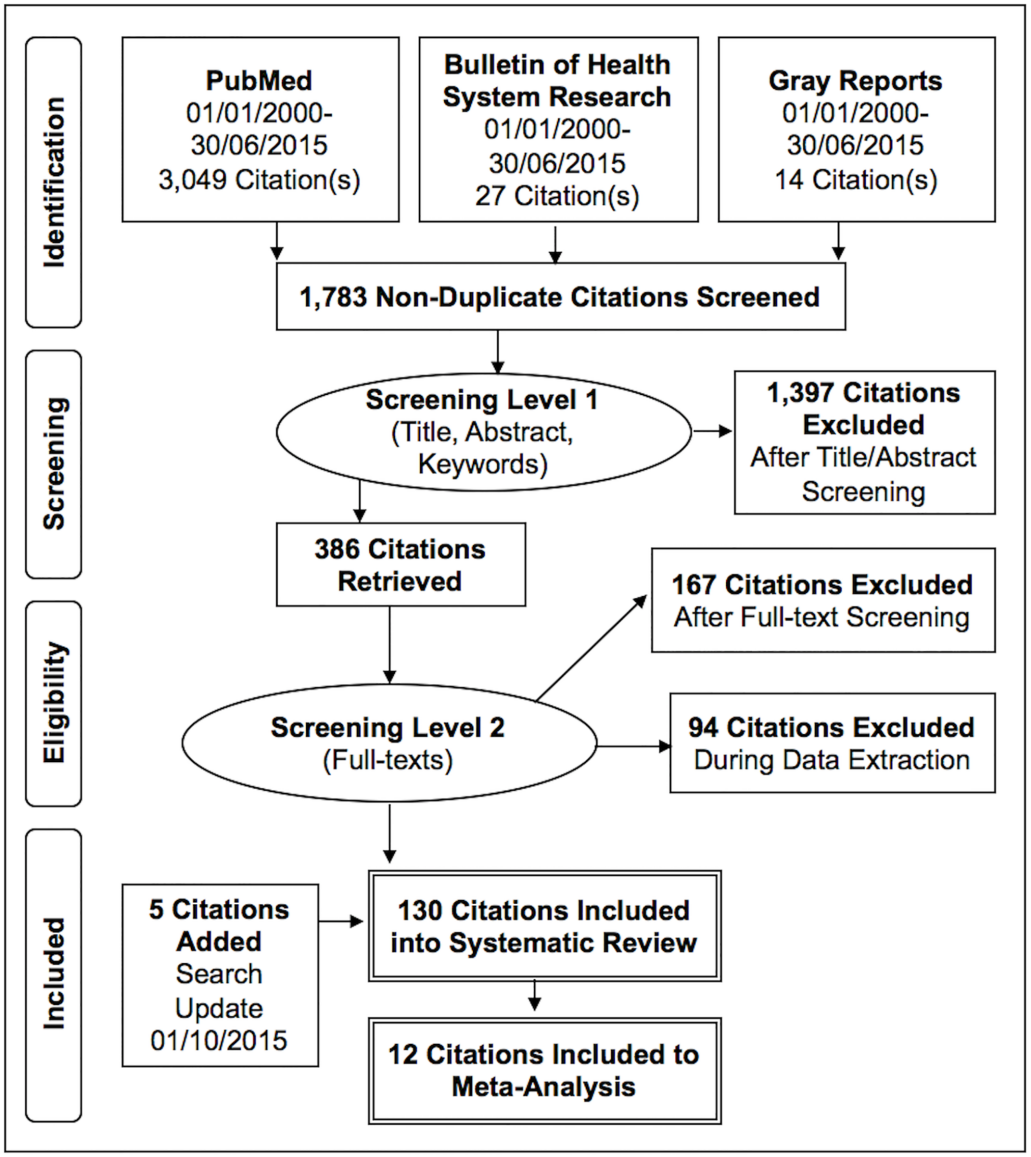
PRISMA flow diagram for bibliographic search.

Our PubMed search returned a total of 3,049 articles. After applying the first level of screening (= titles, keywords, abstracts), 391 articles were identified as potentially relevant. When applying the second screening level (= full texts), 106 articles were identified as closely related and were included for data extraction and analysis [14–119]. From the initial 27 articles identified in the Indonesian journal Bulletin of Health System Research, we included 14 in this review [120–133]. Additionally, 10 out of 14 gray reports were added through hand searches [134–143]. The whole flow diagram presenting the selection process of the final 130 included citations is shown in Fig 1.

### Data extraction

In this work, a synthesis of two common frameworks in social epidemiology served as points of departure: the Disablement Process model by Verbrugge and Jette [144] and the WHO Social Determinants of Health Inequalities model [145]. The first focuses largely on the biomedical process of health change, i.e. from risk factor exposure to mortality via pathology, impairment, function loss, and disability. The latter, however, seeks to integrate evidence that links an interrelated set of social-behavioral components (i.e. structural and intermediary determinants) to explain inequalities and inequities during the disablement process [146]. Guided by these two frameworks, we created and piloted our data extraction form on ten randomly selected articles. We then extracted the following information from each eligible article and report in order to systematically assess the data: first author’s name, publication date, title, study setting(s), year(s) of data collection, study design and analysis methods, sample size including age and sex distributions, disablement process factors addressed in study, type of social determinants of health adjusted for in the analyses, main results or conclusions.

We assessed the quality of quantitative observational studies using the point system from the Newcastle-Ottawa Scale (NOS) [147]. The NOS provides a separate tool for cross-sectional, cohort, and case-control studies. Studies could score a maximum of nine points. A higher point score correlated with a lower risk of bias in the individual studies. Qualitative studies were assessed using the Critical Appraisal Skills Program (CASP) [148, 149]. Qualitative papers can receive the following three categories regarding their quality criteria: “clearly met”, “clearly not met” and “unclear”. Gray reports were evaluated using the AACODS checklist, which highlights the areas of Authority, Accuracy, Coverage, Objectivity, Date and Significance [150].

We analyzed the majority of selected citations using deductive content analysis, which is a widely used research technique in public health [151]. This method is used when the structure of analysis is already operationalized on the basis of an existing framework. This method is able to deal with large textual data and the analysis provides an opportunity to make replicable and valid inferences from data to their context and to construct practical and corroborating evidence to guide policy [152]. Due to the heterogeneity of outcomes and results across studies, many of our results are presented in the form of a narrative synthesis. A meta-ethnography was not meaningful but wherever possible we meta-analyzed data for certain risk factors using a random-effects meta-analysis model. Due to data limitations, we only calculated pooled prevalence (95% CIs) and means (SD) by meta-analysis where at least five studies were available. Heterogeneity among studies was assessed with the Cochrane chi-square (X^2^) and further quantified by I^2^ [153]. Statistical significance was defined at p<0.05. Publication bias was tested by using the Begg and Egger tests. All analyses were done using *metan* in STATA Version 12 (Stata Corporation, College Station, Texas, USA). The PRISMA Statement (Preferred Reporting Items for Systematic Reviews and Meta-Analysis with a focus on health equity) [154] guided the methods of this work. No protocol was registered.

## Results

### Research and bibliometric characteristics

**Fig 2:**
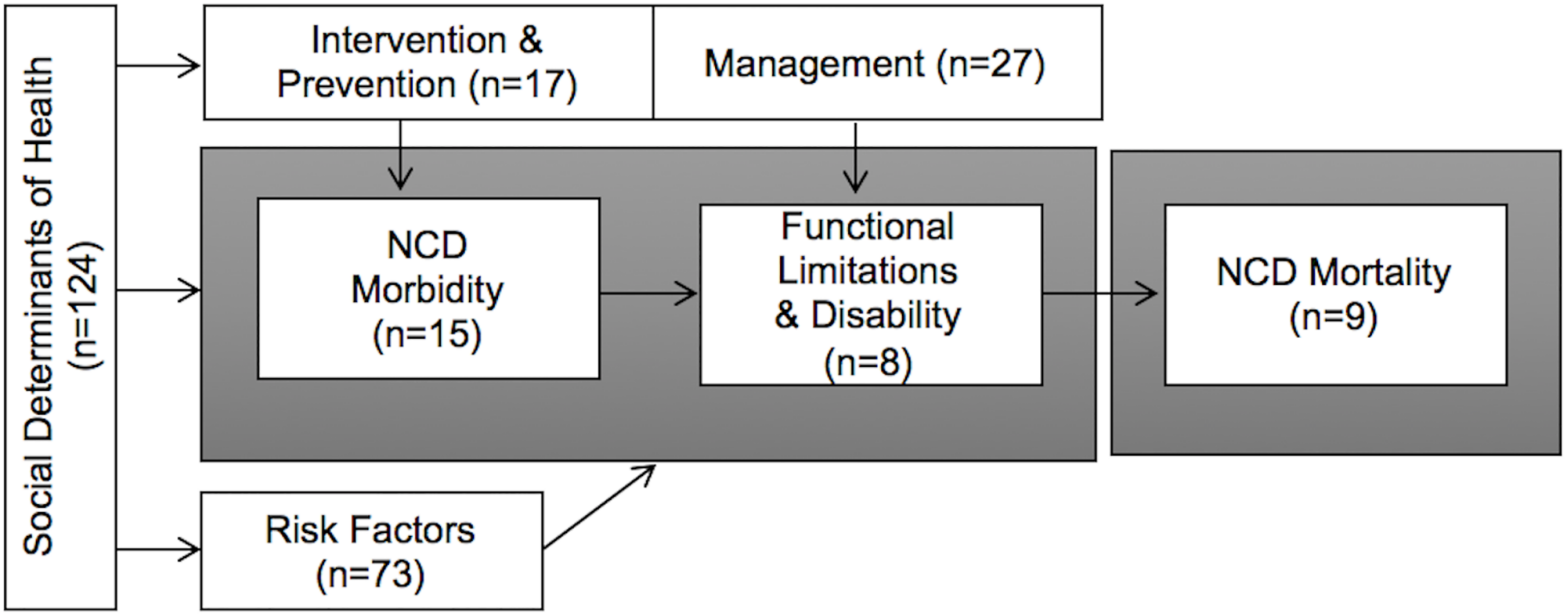
Summary of results showing the number of citations in each domain of the disablement process.

The systematic searches yielded 1,783 nonduplicate citations of which 130 (7%) met our eligibility criteria. All were published between the years 2000 and 2015 using data collected at the earliest in 1989 and at the latest in 2013. The number of data collections as well as publications increased considerably during the past five to ten years, indicating the growing importance of NCDs in public health and epidemiological research in Indonesia. However, while there has been a steady increase in citations using quantitative methods since 2000, it is only in recent years (2010 onwards) that qualitative studies on NCDs have emerged. Of the 130 included citations, nine used qualitative research designs [52, 59, 74, 77, 88, 92, 93, 100, 117], two utilized mixed methodologies [47, 113], and seven focused on the project design or evaluation of risk factor interventions or NCD prevention [30, 51, 57, 61, 65, 78, 86]. Ten of the citations were based on gray reports [134–143]. The vast majority employed quantitative methods (n = 102) [14–29, 31–46, 48–50, 53–56, 58, 60, 62–64, 66–73, 75, 76, 79–85, 87, 89–91, 94–99, 101–112, 114–116, 118–133]. Among these were 84 cross-sectional, ten cohort, and eight case-control studies. Fifty studies performed primary data collections mostly in hospital-based settings. The majority used community-based or population-based secondary data sets such as from the Purworejo Health and Demographic Surveillance Site (HDSS), Indonesian Family Life Surveys (IFLS), National Basic Health Research Studies (Riskesdas), National Socio-Economic Household Surveys (Susenas), or the WHO STEPwise approach to Surveillance (STEPS). Sample sizes ranged from 18 stroke survivors in a qualitative study in Central Aceh [59, 77] to a sample of 664,196 people participating in the 2007 Riskesdas study [127]. The majority of studies included both women and men; a few focused on sex- or gender-specific issues and included only one group. With regard to the authors’ affiliation, in 53% of all cases the first author was based in Indonesia while this applied to only 37% of the last authors. When the first or last authors were not based at an Indonesian institution, more often than not (79%) the author was based in a high-income country such as the USA, Sweden, or the Netherlands. On the basis of deductive content analysis, the 130 included citations were grouped into seven topic areas: (1) risk factors, (2) morbidity, (3) functional limitations and disability, (4) mortality, (5) disease management, (6) interventions, prevention and management, and (7) social determinants of health; an overview of this is provided in Fig 2. A final quantitative synthesis meta-analyzed a subset of 12 studies related to the most common NCD risk factors: smoking (n = 7) [28, 40, 46, 65, 99, 101, 123], hypertension (n = 5) [28, 40, 46, 65, 134], and obesity (n = 7) [15, 28, 35, 46, 65, 67, 119]. Readers please note that a citation could belong to more than one topic area.

### Risk factors

Seventy-three citations focused on behavioral (tobacco smoking, alcohol consumption, physical inactivity, unhealthy diet) and biological (overweight/obesity, hypertension) risk factors for NCDs [14, 15, 18–24, 26–29, 31, 33, 35, 37, 40, 42–50, 53, 60, 62, 64, 65, 67–71, 79, 80, 84, 85, 89, 96–99, 101, 102, 104, 111, 112, 117, 119, 122, 123, 125, 126, 128–140, 142, 143, 155].

The most commonly studied behavioral risk factor was the use of tobacco in the form of cigarette smoking. We found several national estimates for smoking prevalence from different data sources. While these are not directly comparable to each other, they do still confirm the relative high prevalence of smoking (highest recorded prevalence was 36% in 2011 [140]), especially among men (highest estimate was 78% in 2001 [134]) and an increase over time, especially since 2006 [98, 134, 137–140, 142, 143].

There were several studies focusing on the association between smoking and specific chronic diseases such as lung cancer [45, 129], chronic obstructive pulmonary diseases (COPDs) [111], pre-diabetes [96, 130], diabetes [43, 70], undiagnosed diabetes [60], coronary heart diseases [18, 133], cardiovascular diseases [71, 123], reoccurring myocardial infarction [23], and stroke [22]. Some studies focused on specific groups of smokers such as women [125], diabetes patients [47], and physicians [33]. A few studies reported on the use of smokeless tobacco [101], betel quid chewing [89], or more recently the use of electronic cigarettes [112].

Only a very few studies are available on the harmful use of alcohol and its associations with NCDs. According to the 2001 STEPS survey, 77% and 98% of all men and women were abstainers [134]. The latest Riskesdas survey reports a national prevalence of 3% [136] while a subnational study estimates the prevalence of moderate drinking to be 6.3% [42].

Despite being a major behavioral risk factor for a range of NCDs, studies on physical inactivity among Indonesian adults are rare. National data suggest a declining prevalence of physical inactivity from 32% in 2008 [139] to 26% in 2013 [142]. A subnational study in Purworejo HDSS shows that men were more physically active than women (26% vs. 12%) and that occupational and transport-related activities were more common than activities during leisure time [44].

An inadequate and unhealthy diet is strongly associated with a wide range of NCDs and a trigger for further biological risk factors to develop and advance. Only a little information was available on behavioral aspects of food intake patterns and food choice such as fruit and vegetable consumption or unhealthy snacking and convenience food. According to the Riskesdas surveys, the majority of Indonesians (94%) did not consume an adequate amount of fruits and vegetables, which is five portions on seven continuous days. This did not significantly change between 2007 and 2013 [137, 142]. Also the STEP surveys in the Depok community showed a lack of adequate fruit and vegetable intake. Between 2003 and 2006, the number of servings per day increased slightly from 1.9 to 2.1 for fruits but remained the same for vegetables (1.7 servings per day). However, the number of people who ate five servings per day slightly increased from 14% in 2003 to 18% in 2006 [135, 136].

There are several studies on diet-related risk factors such as lipid profiles and body fat distributions [14, 15, 26, 49, 53, 64], trans fatty acids [68, 69], dyslipidemia [49], and metabolic syndrome [19, 27, 37, 62]. Thirteen studies provided insight into the risk factor overweight/obesity [20, 21, 48, 67, 79, 80, 84, 97, 102, 139, 142, 143, 155]. National data estimate that 16–21% of all Indonesian men and 26–31% of all women were overweight; almost 5% of the whole population was obese [97, 139, 142, 143]. We found two more studies focusing on body mass index and diabetes [67] and cardiovascular diseases [132]. One study focused on underweight as a risk factor for coronary heart diseases [80]. We found one study focusing on the increase of overweight and obesity among Balinese women undergoing urbanization and lifestyle changes [21] and three studies focusing specifically on the dual burden of the malnutrition phenomenon, which appears when populations undergo a range of demographic and epidemiological transitions [20, 84, 102]. Two studies focused on the association between overweight/obesity and hypertension [35, 46]. The WHO estimated that in 2008, 37.4% of the whole population had raised blood pressure. The last STEP survey in Depok from 2006 estimates the proportions of raised systolic and diastolic blood pressure to be 23% and 11%, respectively [136]. A total of seven studies focused on blood pressure among varying populations such as urban women [104], rural adults [50], and overweight and normal-weight elderly people [31], and among people without anti-hypertensive medication [24]. Three studies focused on risk factors and social determinants for hypertension [117, 126, 128]. We identified ten studies that focused on several of the above-mentioned behavioral and biological risk factors and their association with NCDs such as stroke [22], coronary heart disease [122, 133], myocardial infarction [23], impaired glucose tolerance, and diabetes [43, 60, 70, 96, 130, 131]. Four studies looked at the frequent co-occurrence of risk factors, so-called clustering [28, 29, 40] or patterns [119].

**Fig 3:**
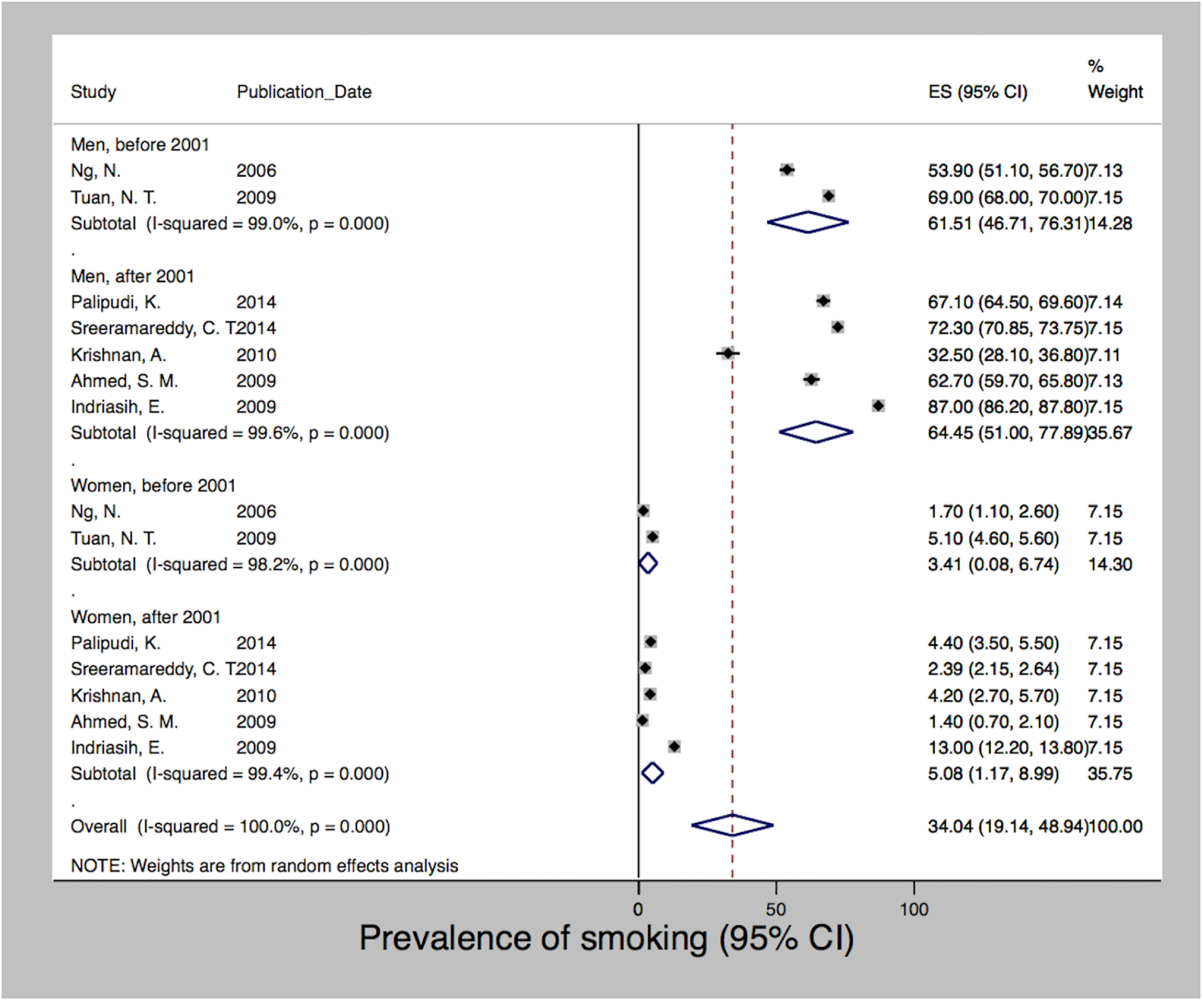
Prevalence of smoking in Indonesia, 2000–2012.

**Fig 4:**
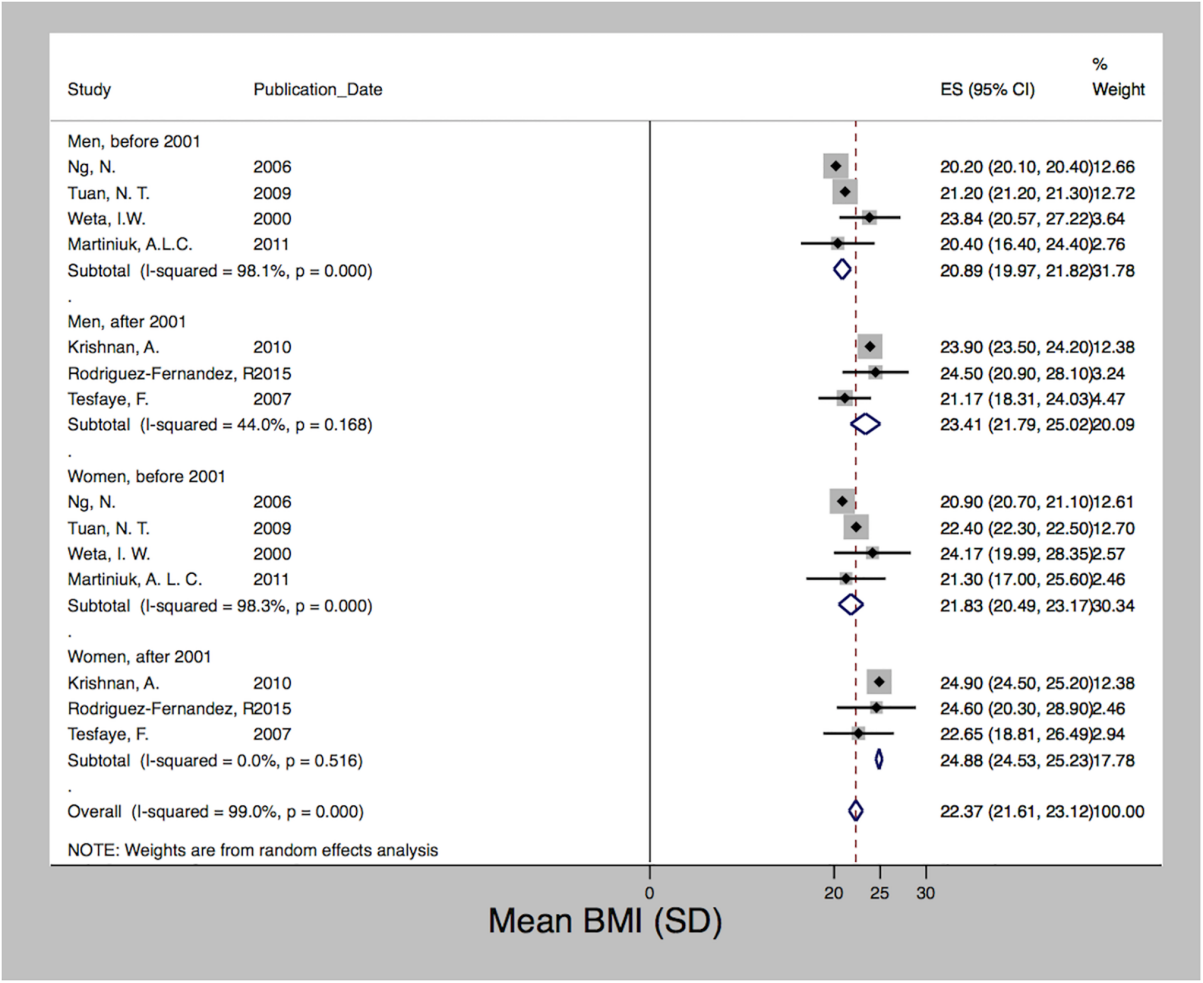
Mean BMI in Indonesia, 1996–2008.

**Fig 5:**
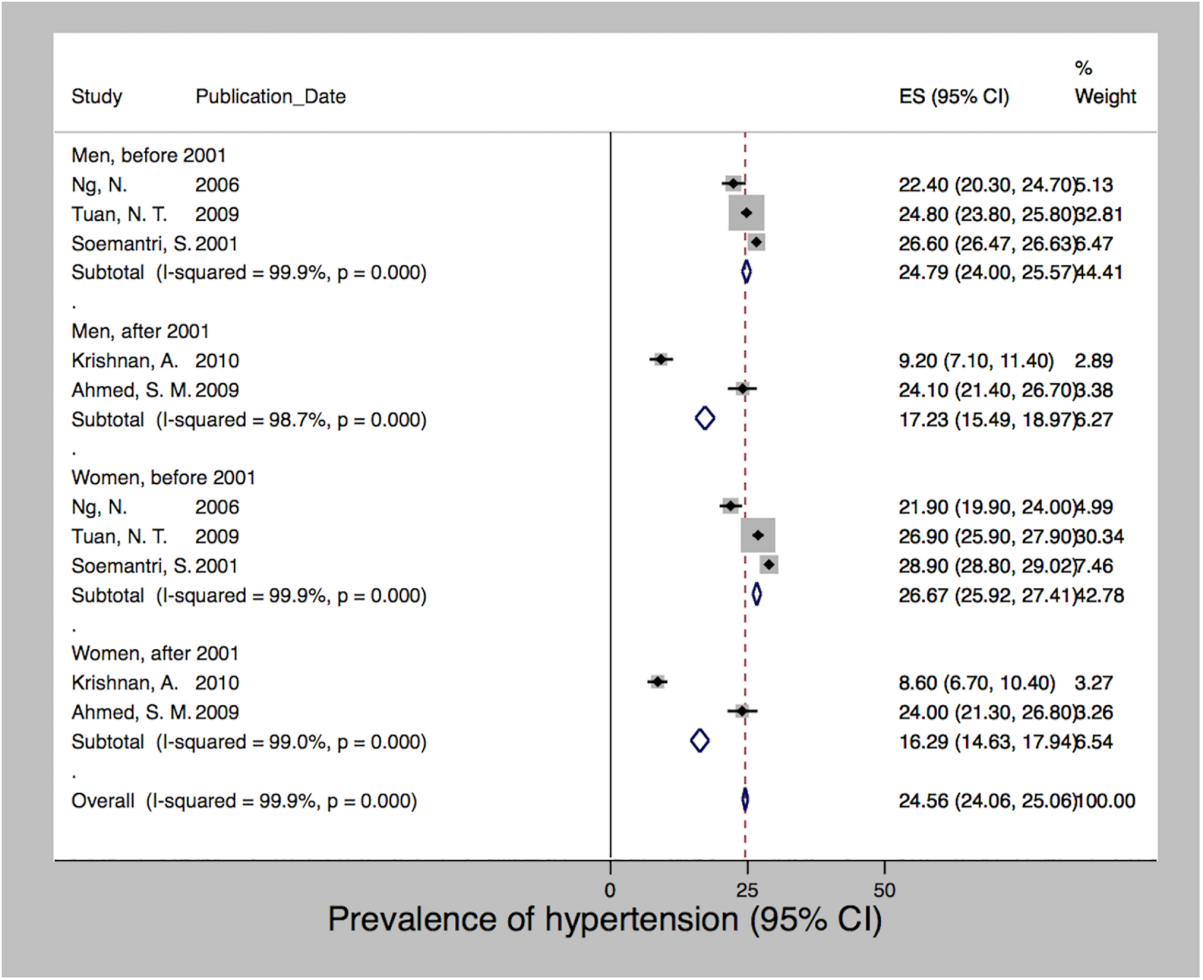
Prevalence of hypertension in Indonesia, 2000–2005.

After meta-analysis of seven studies, which used data collected between 2000 and 2012 [28, 40, 46, 65, 99, 101, 123], we estimated a pooled prevalence of smoking of 34% (95% CI 19.14–48.94). We observed a high degree of heterogeneity (I2 = 100%; p<0.001) with prevalence rates ranging from 1.4% to 87%. Fig 3 shows that the prevalence of smoking differed significantly between men and women. Due to data limitations we were not able to stratify by any other determinants. However, we present the data based on the year of data collection with a cutoff in the year 2001 when Indonesia’s decentralization took place. Decentralization radically transformed the country’s political system, shifting autonomy and decision-making power on key functions of state, including the provision of healthcare, towards the districts. For both men and women, the prevalence of smoking increased over time from 61.5% and 3.4% before 2001 to 64.5% and 5.1% since 2001, respectively. Fig 4 shows a similar trend with regard to obesity, which was derived after pooling seven studies using data collected between 1996 and 2008 [15, 28, 35, 46, 65, 67, 119]. Mean BMI increased following decentralization in 2001 for both men and women from 20.9 to 23.4 and 21.8 to 24.9, but women at all times had a higher BMI than men. The pooled mean BMI was 22.4 (SD 21.6–23.1; I2 = 99%; p<0.001). The estimated pooled prevalence of hypertension was 24.6 (24.1–25.1). Among the five studies [28, 40, 46, 65, 134] that used data from the years 2000 to 2005, we observed a high degree of heterogeneity (I2 = 99%; p<0.001) with rates ranging from 8.6% to 28.9%. In contrast to smoking and obesity, Fig 5 shows that the prevalence of hypertension varied less between men and women and showed a decreasing trend after 2001 for both sexes, i.e. from 24.8 and 26.7 to 17.2 and 16.3 for men and women, respectively. We detected no significant publication bias for hypertension and obesity; however, both the Begg (p = 0.025) and Egger (p = 0.001) tests were significant for publication bias among the smoking studies.

### Morbidity

We found few studies (n = 15) focusing on NCD morbidity compared to the plethora of risk factor studies. The nationally representative Riskesdas studies provide morbidity data based on self-reported physician diagnosis and the former plus a diagnosis based on symptoms [137, 138, 142]. With a self-reported and symptom-based prevalence of 7% and 12%, respectively, stroke was the most frequent chronic morbidity in 2013 [142]. Few studies provide morbidity information about chronic heart diseases. The self-reported prevalence of coronary heart diseases was 0.9% in 2007 and 0.5% in 2013. The symptom-based prevalences were 7.2% in 2007 and 1.5% in 2013 [137, 142]. One study described data on rheumatic heart disease providing an incidence density of 6.84 per 10,000 person years [118]. Morbidity data for chronic lung diseases are likewise limited. Riskesdas reported a COPD prevalence of 3.7% and 4.5% for asthma [142], which is similar to the EPIC Asia study, which reported 4.5% [110]. Another study showed that the prevalence of COPD in nonsmoking individuals from rural and urban Indonesia was 6.3%, of which a significant proportion (94%) were previously undiagnosed [111]. The 2013 Riskesdas study yielded a national cancer prevalence of 1.4% [142]. In addition, we found a few studies reporting morbidity data for nasopharyngeal cancer [72, 87], lung cancer [129], and breast cancer [36]. Several studies provide morbidity estimates for diabetes and impaired glucose tolerance for urban populations [43, 96], and suburban populations in Ternate City [70], and the Bogor municipality [130]. The burden of diabetes varies between Riskesdas 2007 and 2013, being 1.1% and 2.1%, respectively. In urban sites it increased from 5.9% to 6.9% [137, 142]. Seventy-two percent of all diabetes cases in 2013 remained undiagnosed [60].

### Functional limitations and disability

Eight studies focused on functional limitations and disability [32, 34, 55, 66, 114, 121, 124, 127]. Nationally representative data from the 1993 and 1997 IFLS show an increasing prevalence of ADL limitations (4.2 to 6.5%) and Nagi’s physical functioning tasks (45.9 to 58.6%) among adults over 60 years of age [34]. Subnational data among adults aged over 50 mirror the rising burden of poor functional limitations and disability. The number of people being categorized in the worst WHO Disability Assessment Schedule (WHODAS) quintile increases with age, especially among women [55]. The most commonly used indicators of functional limitations were the Activities of Daily Living (ADLs) and Instrumental Activities of Daily Living (IADLs) [66, 114], Nagi’s functional limitations [32], or a combination of all [34]. Three studies employed the International Classifications of Functioning, Disability and Health (ICF) [121, 124, 127] and one study the WHODAS [55]. Most studies focused on middle-aged and older adults [32, 34, 55, 66, 114] and three on the general adult population aged more than 15 years [121, 124, 127].

### Mortality

Death registration systems and cause of death ascertainments are rare in Indonesia; only nine studies provided data on NCD mortality. Up until 2006 Indonesian mortality measures were derived from intermittent household surveys and estimates were based on indirect demographic life table methods approximating mortality patterns in neighboring countries. The most comprehensive estimates are from the WHO and outline clearly that the number of deaths attributable to NCDs increased between 2008 and 2012 from 64% to 71%, respectively, with cardiovascular diseases (37%), cancers (13%), diabetes (6%), and chronic respiratory diseases (5%) being the leading causes of death in 2012 [139, 143]. The Global Burden of Disease (GBD) estimates additionally highlight that the rates of premature deaths were significantly higher for cerebrovascular disease, diabetes, and asthma [141]. A recent update of the IDF Diabetes Atlas attributes 386,400 deaths to diabetes in Indonesia in 2013 [116]. Stroke is the leading cause of death with rates of over 300/100,000 [82]. One study provides estimates of lung cancer deaths, which exceed the WHO estimates by 1.24 times, presenting a crude lung cancer mortality rate of 17.6 per 226,063 people [45]. Results from the Indonesian mortality registration system indicate that stroke, diabetes, and ischemic heart disease are the most prominent causes of death in urban Surakarta, accounting for 27%, 8.5%, and 7% of all deaths during 2006–07. In rural Pekalongan, stroke (19.9%), other heart diseases (7.5%), and chronic respiratory diseases (7.1%) were the leading causes of death [61]. We found two studies on the association between self-rated health and mortality [17, 76]. The predictive power of poor self-rated health for subsequent mortality held even after controlling for nutritional status, physical functioning, symptoms of poor physical health and depression [17], and education and household socioeconomic status [76].

### Disease management

Through our review we identified 27 citations that dealt with NCD management at the structural policy level [25, 61, 88], clinical level [23, 54, 63, 72, 75, 83, 90–92, 95, 106, 108, 110, 113], community [16, 38, 52, 73, 74, 100], family [105, 107], and individual level [59, 77]. At the structural level, we recognized three issues: health insurance services for NCD patients [88], the need for a better collaboration between health and registration sectors [61], and the need to address the double burden of NCDs and infectious diseases such as tuberculosis and diabetes [25], which might be aggravated by smoking [39].

At the clinical level, we identified clear needs to improve the management of cancer, i.e. nasopharyngeal cancer [54, 72], breast cancer [83, 91, 92], cervical cancer [108], and colorectal cancer [75]. Tackling low screening uptake [75], improving early diagnosis, and increasing compliance with treatment [72] as well as better advanced physician’s knowledge and continuous education [54] are necessary steps to be taken. One study identified the heightened need for physicians to be aware of patients’ unrealistic beliefs in delaying treatment and consulting traditional healers [83, 91, 92]. One study raised problems and unmet needs in relation to palliative cancer care [106]. With regard to hypertension and chronic heart diseases, the studies that we found indicate clear needs for improving the overall care for myocardial infarction [90], to manage overall unmet needs for cardiovascular care [95], provide appropriate medication for recurrent coronary heart and cardiovascular diseases [23], and to address the lack of urgency among doctors to control hypertension [113]. The clinical management of chronic lung diseases and diabetes was addressed by two studies, which both called for improved physician education in the field of COPD [110] and diabetes [63].

There has been only a little research on how the community may be involved in the successful management of NCDs. The role of involving the community in diabetes management has been addressed by three studies [38, 73, 100]. We found two studies on community perceptions and awareness of stroke and CVD [16, 52] and one on community mobilization for cervical cancer treatment [74]. Little is available about chronic disease management at family level. Only Effendy and colleagues provide insights into the involvement of family caregivers in cancer care [105, 107]. At the individual level, there has been some research on the individual perception and impact of stroke [59, 77].

### Interventions and prevention

Despite a high burden, efforts to intervene or prevent NCDs and their risk factors are limited. We identified 17 studies that dealt with NCD-related interventions, including prevention and management strategies. Most addressed tobacco and smoking prevention [30, 56–58, 81, 93, 94, 109, 120], followed by CVDs and hypertension [51, 86], diabetes [103, 115], and cervical cancer [78]. The “Quit Tobacco International” project [57], the “Program to Reduce Cardiovascular Disease Risk Factors”, PRORIVA [51], and a “See and Treat Program” for cervical cancer [78] were the most prominent programs and interventions cited in the literature. General integrated NCD and risk factor interventions for prevention were addressed by the WHO STEPS surveys [65, 135, 136]. Besides focusing on the general population (e.g. [51, 65, 86]), some used special groups such as physicians [30, 103] or pharmacists [94, 109, 115, 120] as facilitators. This was especially the case when preventing and intervening the rising burden of tobacco smoking and diabetes. One fact that clearly points out a disadvantage of rural communities in terms of being underserved by prevention and intervention programs is that all programs found in the literature were conducted in Jakarta [78], Yogyakarta [30, 51, 56–58, 86, 94, 109], or a combination of major cities [81, 93, 103, 115, 120].

### Social determinants of health

**Fig 6:**
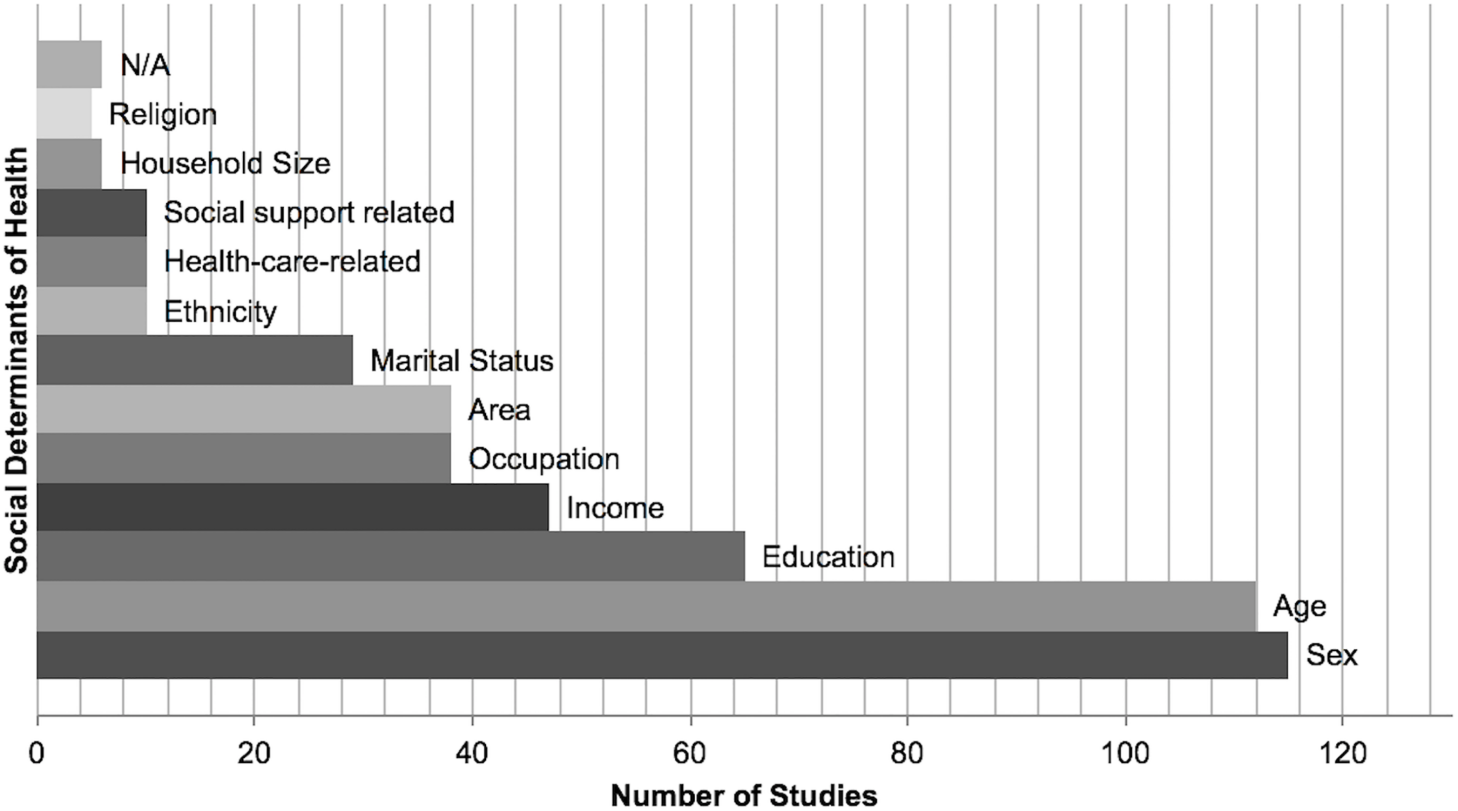
Frequency and types of social determinants used in 130 citations.

Fig 6 shows that most of the 130 included studies considered age (n = 115) [14–17, 22–29, 31–50, 53, 55, 56, 59–64, 66–73, 75–77, 80–92, 95–102, 104–108, 110–129, 131, 133–143, 155, 156] and/or sex (n = 112) [15–19, 21–26, 28, 29, 31–38, 40–50, 53, 55, 56, 58–92, 95–108, 110–112, 114–116, 118–123, 125–129, 131, 133–143, 155, 156] in their analyses. On average around 40% of all studies included some sort of socioeconomic indicator mirroring educational attainment [15, 17, 21–24, 32, 34, 35, 37–40, 42–44, 47, 48, 50, 55, 56, 60, 63, 66, 68, 69, 71, 73, 75, 76, 79, 81, 83–85, 91, 92, 95, 99, 101, 102, 104–108, 111, 112, 114, 122, 123, 125–127, 129, 131, 133, 135–138, 140, 142, 155, 156], income or wealth [15, 17, 20–22, 25, 28, 37, 39, 43, 47, 52, 55, 56, 60, 63, 66, 71, 75–77, 79, 84, 86, 91, 95, 96, 99, 101, 102, 104, 111, 112, 114, 123, 125–127, 131, 133, 135–138, 142, 155, 156], and occupational status [15, 24, 33, 35, 43, 47, 69, 73, 74, 83, 84, 88, 91, 92, 103, 104, 107, 108, 110, 111, 113, 115, 118–120, 122, 123, 125, 127, 129, 131, 135–138, 140, 142, 155]. About the same proportion of studies contained information on people’s place of residence such as whether they reside in rural or urban areas or the province in which they live [14, 17, 20, 28, 31, 32, 35, 46, 61, 66, 68, 71, 72, 76–79, 81, 84, 88, 93, 95, 99, 100, 102, 111, 114, 122, 124–128, 134, 137, 138, 140, 142]. Despite the fact that Indonesia is home to a great number of indigenous and spiritual groups, ethnicity [21, 64, 72, 83, 89, 104, 131, 133, 135, 136] and religion [32, 38, 101, 104, 117] were less frequently applied in analyses. Only a few recent studies adjusted their analyses for access to healthcare-related factors such as access to services or insurance status [60, 63, 75, 83, 87, 88, 91, 92, 95, 133]. We found several studies on determinants that go beyond the individual level such as marital status [14, 22, 32, 34, 38, 39, 41, 55, 56, 58, 73, 76, 79, 83, 95, 101, 104, 105, 108, 114, 117, 125, 126, 131, 133, 137, 138, 142, 155] or household size [20, 38, 55, 66, 84, 136]. However, facilitating other cross-cutting determinants such as community social capital [102], social support [38], or social participation [114] is still infrequent but has increased in recent years.

## Discussion

This is the first work to systematically review and synthesize scientific NCD-related evidence for Indonesia–a country hosting the largest population in Southeast Asia and the fourth largest worldwide. Logistically, our review emphasizes the benefits of facilitating heterogeneous data, methods, and research designs that may be used to research NCD-related evidence in Indonesia including their progression, advancement, and growing public health significance over the past decade. In addition, we highlight the growing and disproportionate burden that NCDs and their risk factors inflict on the country, in particular pointing out social inequalities in NCD outcomes and exposures but also in care and treatment, which are major barriers in the reduction of the total burden of NCDs on the way to achieve good health for all.

Specifically, we offer three main findings from this review. First, our findings echo the urgent need to expand routine NCD risk factor surveillance and outcome monitoring such as cause-specific morbidity and mortality information and to better integrate these into one national health information system to support evidence-based decision-making. Second, there is a stringent necessity to reorient and enhance health system responses to offer effective, realistic, and affordable ways to better prevent and control NCDs through priority and cost-effective interventions. Further on that note, our results maintain the call for a more structured approach to the delivery of primary NCD care and to further develop and coordinate the use of high-quality primary and clinical care services for the delivery of more equitable interventions and treatment strategies for NCDs. Third, our review strongly suggests expanding research on social determinants of health as well as strengthening the research to policy link, specifically for cross-cutting determinants beyond the individual level to the extent that a reduction of the total burden of NCDs and NCD inequalities should be looked upon as an interrelated and mutually reinforcing priority.

### Strengthening health information systems to support NCD programs in Indonesia

By illustrating the health burden attributable to NCDs, we found that there is a strong discrepancy with regard to how much data–but also research–is available and published per each topic area. Risk factor studies are manifold (n = 73) but studies on morbidity (n = 15), functional limitations and disability (n = 8), and mortality are infrequent (n = 9). This echoes the need to expand routine outcome monitoring while keeping up periodical risk factor surveillance.

Monitoring risk factors at population level has been a mainstay of NCD surveillance in many countries. To date, 132 LMICs have applied the WHO STEPS method for risk factor surveillance [157, 158]. Indonesia is among the few that have conducted several surveys, namely a national STEPS survey in 2001 and subnational surveys in Depok in 2003 and 2006 [65, 134] and Purworejo in 2001, 2005, and 2010 [28].

As this review has demonstrated, up until 2006 Indonesian mortality measures originated from intermittent household surveys and estimates were derived through indirect life table methods approximating mortality patterns in neighboring countries [159]. Indonesia’s government mandated compulsory death registration across the country by enacting the Population Administration Law in 2006 [160]. Citizens need to report a death within 30 days to the *kelurahan* (urban administration office) or the *desa* (village administration office) along with some supporting documentation from a health professional. To further improve the quality of death registration and cause of death ascertainment, the Indonesian Mortality Registration System Strengthening Project has advocated the use of verbal autopsy reports. However, in the aftermath of decentralizing the health system in 2001, inadequate registration and intersectoral collaboration between registration and health departments remain a challenge on the way to reaching international standards [61, 161]. A technical review of the Indonesian Health Information System from 2007 shows the inadequacy of the assessment of health status indicators (mortality, morbidity) and risk factors but still points out the high demand for evidence-based decision-making by executive or legislative sectors [162]. Since 2010, a joint decree between the Ministry of Home Affairs and the Ministry of Health has contributed to further development of a mortality registration system in Indonesia in 128 randomly selected subdistricts covering eight million people in 2013 [163].

Morbidity and disability information is important for policy and program development, but despite overwhelming needs, only small parts of the population in Asia are being monitored. Indonesia is one of the few Asian countries without a national population-based cancer registry [164] or a central diabetes register [165], and it also has no surveillance and monitoring system in place to enable reporting against the nine global NCD targets including the “25 by 25” goal [166].

Current means to strengthen and centralize the Indonesian health information system and to support the inclusion of NCD programs need further determination. Disease and risk factor surveillance so far has not become institutionalized as a vital public health function and has built up little sustained country capacity. Acceleration of financial and technical aid is likewise required for the country’s health information system to develop.

### Expanding Indonesia’s health system responses to NCDs

By illustrating existing approaches for intervention, prevention, and disease management, we found that the way Indonesia’s health system responds to the rising number of NCD patients still resembles the individual-based and infectious disease-oriented approach that set the agenda for many decades. We found some studies on prevention and intervention activities (n = 16) with the majority focusing on the integrated risk factor interventions within the STEPS surveys [65, 135, 136] or on smoking prevention [30, 56–58, 81, 93, 94, 109, 120].

The WHO deemed salt reduction and tobacco control to be high-priority cost-effective interventions for achieving the “25 by 25” goal [167]. Our review showed that salt consumption has not been addressed rigorously by any study; only one measuring sodium intake [31] and intake of foods with high salt, monosodium glutamate, or flavors is reported in the Riskesdas studies [142]. In LMICs, mass education to encourage salt reductions and regulatory measures in the food industry has been proven to work [168] but efforts in Indonesia have not yet made their mark. Recently, a salt module was added to the STEPS survey instrument [157] but this has not yet been implemented in Indonesia. The Indonesian government currently has six regulations related to food in place, two of which are directly related to salt intake. However, challenges such as receiving valid information about salt intake and intervening in the industry sector remain [169]. The meta-analysis results on the prevalence of hypertension showed a decrease in hypertension after 2001; due to data limitations we were unable to link this to salt intake, i.e. in the form of a meta-regression. Future studies are needed to study salt intake and to understand how policies can best be used to promote salt reduction initiatives.

Today, Indonesia has the fifth highest tobacco consumption rate in the world and spends 1.78 billion USD on tobacco-related healthcare while the annual spending on cigarettes by Indonesian smokers is six times the average expenditure on education and health [170]. Despite this tobacco epidemic, Indonesia remains the only country in the Southeast Asian region that did not ratify the WHO Framework Convention on Tobacco Control (FCTC) [166]. Our review identified smoking as the most frequently studied behavioral risk factor. The results from the meta-analysis confirmed the high prevalence and an increasing trend over time both among men and women. In the absence of any national tobacco control legislation or directives, there are still other strategic actions that we identified in this review including the development and dissemination of standard guidelines for cessation services for use by health professionals and providing technical support in training health professionals in tobacco cessation. The Quit Tobacco International program [30], which supported physicians, most distinctly exemplified this along with other smaller studies using pharmacy students (e.g. [94]) as cessation facilitators.

Evidence on disease management was more frequent (n = 29) and echoed the necessity for a health insurance provision guaranteeing more equitable and affordable treatment strategies including a reorientation of the health system towards the provision of structured, high-quality, and long-term care. For more than a decade now Indonesia has been in the process of ensuring effective decentralization and a well-functioning health system while at the same time encountering pressing health needs brought about by natural disasters as well as emerging and re-emerging communicable and noncommunicable diseases. While decentralization clearly imposes new challenges it also presents new opportunities. Local governments have become the focal point for healthcare provision with their share in total public health spending increasing from 10% prior to decentralization to 50% in 2001 [171]. This shift could make public spending more responsive to local conditions and variations in disease patterns towards NCDs. The Ministry of Health has recently announced a set of health system reforms (BPJS) aimed at attaining universal health coverage by 2019 by progressively covering 140 million uninsured citizens through the national health insurance schemes *Askeskin* and *Jamkesmas* [172]. BPJS has also developed guidelines for NCD program management for diabetes and hypertension [173]. Despite its significance, our review identified only very little evidence relating to health insurance coverage among patients with chronic illnesses (e.g. [88]), which may be due to the very recent introduction of *Askeskin* and *Jamkesmas*. Nonetheless, there is clearly a need for further research in terms of use and its determinants, provision, or satisfaction and economic hardships. The process towards UHC–and health insurance history in general–has been described as uneven and iterative, however, driven by domestic political interests to a degree that it always tried to meet the political priorities of the day [174]. It is yet to be seen how future insurance schemes will respond to an aging population confronted with rising healthcare costs due to chronic disease and disability.

We identified a need to improve the structural management and quality of NCD care, specifically pointing out continued physician education including specializations in chronic diseases, familiarization with long-term and palliative care, and fostering a mindset that makes doctors understand the urgency to control “silent” conditions such as diabetes or hypertension and deliver preventive messages on, but not limited to, smoking cessation and dietary counseling. Our review likewise showed that due to the scale of the burden of NCDs and the cost implications, evidence points towards the importance of a primary care-centered response to NCDs rather than hospitals at secondary or tertiary levels [175]. Primary care’s key strength is that it is the main entry point to health services and it previously played a good key role in the delivery of key interventions for tuberculosis, HIV, and malaria in many low-resource settings [176].

Like many other LMICs, Indonesia’s health system approach to NCDs is still very much on an individual basis but it has been shown that a public health approach with a programmatic structure, systematic follow-up, and monitoring of quality of care and routine outcome reporting is much more beneficial in such settings [177]. The benefits of shifting from an individual approach to a public health approach have been exemplified by the experience of ART for people with HIV [178]. However, these lessons have not yet been applied to people with NCDs, not just in Indonesia [179].

Indonesia’s approach to the delivery of care is also focused on the management of single diseases as opposed to multiple morbidities. Evidence on multimorbidity is scarce, but with rising NCD prevalence and life expectancies this burden will likewise increase and greater focus on the management of comorbid conditions is needed. Indonesia has recently adopted WHO PEN (Package for essential noncommunicable disease interventions for primary healthcare in low-resource settings) [180]. A comprehensive generalist care approach that is mainly focused on but not exclusive to primary care has been suggested as the most suitable strategy for the management of people with multiple morbidities. However, any implementation of new guidelines needs to be complemented with studies to assess operational challenges in the delivery of care [181].

While monitoring exposure and outcomes illustrates the burden of NCDs, monitoring health system responses mirrors a country’s capacity to address, prevent, and control NCDs. Indonesia’s struggle to develop a responsive healthcare system is intensified by an environment where health insurance coverage is incomplete and where a structured approach to the improved delivery of primary NCD care is lacking. Indonesia is facing a complex double burden of disease so that despite a rising burden of NCDs, the majority of health interventions still focus on reducing high child and maternal mortality and malnutrition. Further, the health system remains largely tailored to acute infections as well as out-of-pocket payments and is not yet fully accustomed to providing high-quality long-term chronic care. On the positive side, many have outlined that the removal of financial barriers to healthcare is an essential component of any credible effort to reduce NCDs and NCD inequalities [182, 183]. In this respect, future research should focus on monitoring and evaluating the ongoing introduction of the BPJS reforms and universal health coverage. Any effort to strengthen Indonesia’s healthcare system towards NCDs should however happen with cooperation between the Ministry of Health and other government ministries (e.g. finance, agriculture, education) and multi-stakeholders as there is great cross-ownership over issues like salt reduction and the introduction of UHC.

### Addressing NCD-related social determinants in Indonesia

By illustrating how and why an inequitable distribution of the NCD burden can be explained in terms of the social determinants of health framework we found that there is a large disparity in terms of how well certain determinants were explored in the Indonesian context. We found plenty of evidence on age (n = 115) and sex (n = 112) as well as indicators of socioeconomic status (n = 82) but determinants that go beyond the individual level appeared less frequently. This echoes the demand to close the knowledge gap between NCDs and additional structural determinants and socioeconomic position such as gender, ethnicity, or sociopolitical context as well as intermediary determinants focusing on material circumstances, social-environmental, or psychosocial circumstances, or the health system [145, 146].

In particular, cross-cutting determinants that go beyond the individual level and touch upon certain contextual specificities such as social capital or social networks have been proven to be highly relevant and beneficial for people with NCDs but remain nearly untouched both in the Indonesian context and in other developing settings [184, 185]. The doctor-patient relationship prevails and is almost exclusive when it comes to treating infectious diseases. However, with the rising burden of NCDs, exploring individual sources of support and collective processes and mechanisms that look at intermediate or mezzo-levels between the health system and patients’ including families’, friends’, or the community’s involvement in NCD prevention or management stand as research and policy needs that need to be addressed in the future [186, 187].

Emphasizing its potential to generate health equity and well-being among both individuals and communities, social capital has been identified as a key social determinant of health [146]. However, its application, particularly in LMICs, remains a challenge [188]. For Indonesia, however, applying a social capital lens to NCDs is particularly of interest against the background of a shift from individually based approaches to health towards a focus on community development and empowerment [86]. As outlined above, there are currently efforts to move towards a public health approach towards NCDs in Indonesia. A prime example is the setup of the so-called *Posbindu PTM*, an integrated health post for NCDs where the community plays a central role in health education and promotion as well as NCD and risk factor prevention [65].

In line with this, social network approaches to NCDs in developing countries likewise have great potential and have been identified as powerful leverages for health interventions [185]. It has long been recognized that resource transfer through personal networks may be most important in environments with limited institutional, state-based, and private sector support. Due to the long-term debilitating and disabling nature of NCDs and the lack of formal social protection schemes in many LMICs, sources of formal support are in many cases not available when needed. Thus, informal support networks from family, friends, or the wider community may be people’s only source of support when it comes to maintaining health and well-being. A systematic review on social networks and health in LMICs points out the lack of in-depth studies on social networks and health in LMICs specifically looking at health interventions and evaluations [189]. Indonesia, despite globalization and fast economic development, remains one of the most collectivist cultures and offers unique opportunities to study the role of social networks in health and vice versa.

On a higher level, the development and implementation of multilevel intersectoral and multisectoral networks and collaborations to build solutions for NCDs beyond the health sector has likewise been a challenge in Indonesia. Given the fact that inequalities in NCDs manifest themselves in the form of differential health consequences due to varied exposure, social stratification, and differential vulnerability, action is needed from health and allied sectors (e.g. education, trade, agriculture, urban planning), as well as civil society and private sector engagement [10, 167]. Such multilevel intersectoral and multisectoral networks play an important role in addressing the multifaceted needs of chronic disease prevention and intervention [190]. This is especially pertinent with regard to developing national responses to the NCD-related targets included in the SDGs [191]. Our review has outlined that the traditional disease-focused health system was primarily designed to respond to acute infectious diseases and is ill equipped to deliver complex, integrated intersectoral, multisectoral, and sustained activities to adequately respond to the challenges NCDs impose on the country. An interview study with 13 Indonesian health policy workers on the dynamics of NCD control policies [192] showed that insufficient political interest in public health, health promotion and disease prevention, disconnected policies, and a lack of institutional arrangements and resources to work under a multisectoral arrangement were key challenges to NCD control in Indonesia. Building up networks of public health experts and policymakers, increasing the understanding of nonhealth sectors of their role in NCD prevention, and not perceiving NCDs as a strict health sector domain (e.g. by offering economic, industry, or environmental analyses of health-related proposals) could be part of the solution. Moreover, Indonesia has successful well-evaluated health promotion examples already in place that could be scaled up or reoriented to include NCDs (e.g. including diabetes in TB programs [165]). In general, implementing a range of health in all policies, whole-of-government and whole-of-society approaches for addressing NCDs as well as setting national targets to develop and strengthen multisectoral policies and plans for NCDs will be necessary steps to take in the future. However, this again relies heavily on the country’s potential to allow evidence-based decision-making driven by a functioning health information system and robust data.

### Strengths and limitations

Our systematic review has several key strengths. Our search strategy was comprehensive, covering international and national electronic databases with no language limitations with regard to English and Indonesian publications. The eligibility criteria were well defined and carefully applied to each study and the review itself evolved along the PRISMA guidelines [154]. A further asset of this work is the extensive amount of data reviewed, representing wide geographical coverage and 15 years of research originating from mixed research designs.

However, the results of this study may be affected by several limitations. We may have overlooked citations that should have been included in this review. This concerns, for example, journal articles published by Indonesian nonelectronic journals hosted by universities. However, we deem it unlikely that important relevant studies, which could potentially alter the conclusions of this review, have been missed. Further, despite growing concern and a rising burden, we did not include pediatric NCDs or mental health conditions in this review but focused only on four major NCD groups. We are aware of the possibility that our findings may be vulnerable to different sources of bias such as the quality of existing evidence and reporting bias, but overall we consider this a very small possible limitation. The mean NOS score of all quantitative studies was 6.48 out of 9 and all qualitative studies clearly met the CASP criteria. We deem this a relatively strong working basis and believe there is only a small risk that poor studies affected our review findings. Publication or reporting bias–the fact that negative results may not be published or only selectively reported–is a major issue in all literature reviews leading to under- or overestimations of effects. We exclude delayed publishing (also known as time lag or pipeline bias) and location bias (including location and language bias) as potential reasons to introduce reporting bias into our study since we cover a 15-year period and both English and Indonesian publications. We used the Begg and Egger tests to statistically test for publication bias, which was significant for the smoking studies but not for hypertension or obesity. A possible reason, besides pure chance, could be the true heterogeneity between the studies resulting from large differences in the prevalence of smoking among Indonesian men and women.

### Directions for future research

The current WHO country cooperation strategic agenda (2013–2017) entails the strategic priorities to promote public health approaches to the prevention and control of NCDs, specifically to support the monitoring of the prevalence of NCDs and related risk factors, to support the implementation of best practices in tobacco control, and adherence to and implementation of the WHO Framework Convention for Tobacco Control, and to support prevention and health promotion to control and prevent NCDs [193]. Addressing these issues with improved coordination of programs and policies that also target poverty-related risk factors, multisectoral collaborations outside the health sector, and accompanying efforts with accountability mechanisms has also been reinforced by the Action Plan for the Prevention and Control of Noncommunicable Diseases in Southeast Asia, 2013–2020 [194]. Our review points out that there are significant developments in place that are directed towards these objectives. However, we also identified gaps and needs that need to be addressed further. These, however, present opportunities for future research and we propose three areas for advancement. First, we propose that future surveillance and data collections shift from repeated cross sections and their inadvisability to make causal claims towards the collection and advanced analysis of longitudinal and panel data with repeated measurements for strengthening health information systems for decision-making in LMICs. Such data and methods will allow exploitation of both inter-individual differences and intra-individual dynamics over time, permit more accurate inference of model parameters and controlling for omitted variables, and imply causal directions. Second, we advocate the adoption of an integrated care approach to NCDs. The WHO defines integrated care as a concept connecting the delivery, management, and organization of all services related to diagnosis, treatment, care and rehabilitation, and health promotion. We suggest that future research should involve multidisciplinarity borrowing methods, theories, and frameworks from disciplines other than primary care or public health. Issues like functional limitations, disability, and multimorbidity, but also rehabilitation and recovery, should become key research areas in order to inform about the adoption of integrated care into health systems in LMIC settings. Focusing not just on the social epidemiology of health conditions but also on the experience of disease at the individual level will offer more holistic insights into how and why diseases cluster unequally within different population groups. Such a “syndemic” approach will aid the understanding of how social and cultural factors shape people’s experiences of morbidity and comorbidity while taking into account how social and structural factors co-occur with certain disease clusters within and between groups. Attending to such a big picture will eventually lead to more successful public health interventions and prevention [195]. Further, provision of care, either the formal organization of it through setting up chronic long-term condition management programs or informally through family caretakers, should become a research focus in the years to come as this is predictably a rising concern and demand in the country. Focusing on how to best organize and integrate such services at healthcare system level or on how individual social, cultural, or economic conditions affect those receiving or providing care will be crucial. Third, despite obvious constraints such as moving towards concepts and measurements that are external to the direct scope of traditional public health research, we advocate more social network studies involving health, in particular among subpopulations such as elderly and chronic disease populations, in LMIC settings in order to explain how the quality and quantity of individual networks affect health and vice versa. Specifically, studies that increase understanding about causal effects of networks on different health outcomes, the role of network characteristics along disablement or recovery pathways, the implications of network changes over time, or loneliness are scarce. Strengthening this research area with quantitative, qualitative, and mixed-method approaches will provide powerful leverages for health interventions and show flashpoints for social and public policies.

## Conclusion

The present work makes an important contribution to the field of chronic disease epidemiology and public health in Indonesia. Our findings echo an urgent need to expand and advance routine NCD risk factor surveillance and outcome monitoring and to better integrate these into one national health information system. There is a stringent necessity to reorient and enhance health system responses to offer effective, realistic, and affordable ways to better prevent and control NCDs through cost-effective interventions and more structured and integrated approaches to the delivery of high-quality primary care and equitable prevention and treatment strategies. Research on social determinants of health, especially on cross-cutting determinants such as social capital and social networks, and policy-relevant research need to be expanded and strengthened to the extent that a reduction of the total NCD burden and NCD inequalities should be treated as related and mutually reinforcing priorities. Developing research on social determinants goes hand in hand with the premise of data availability but also the advancement of theories and methods to support multidisciplinary research. This review offers a systematic and comprehensive investigation of the burden that NCDs inflict on the country, which will be valuable for health policy and planning and guiding future research.

## Supporting information

S1 File Search syntax for PubMed database. (DOCX)

S1 Checklist PRISMA checklist. (DOC)
