# Supplementary material for: How is Indonesia coping with its epidemic of chronic noncommunicable diseases? A systematic review with meta-analysis
Source: PLoS One. 2017 Jun 20;12(6):e0179186. doi: 10.1371/journal.pone.0179186 (PMC5478110; doi:10.1371/journal.pone.0179186)
Supplement: S1 File — (DOCX) [file pone.0179186.s001.docx]

**Supplementary Information.**

**Schröders et al. 2017. “How is Indonesia coping with its epidemic of chronic noncommunicable diseases? A systematic review with meta-analysis”**

We used the following combination of terms in PubMed (<https://www.ncbi.nlm.nih.gov/pubmed/)>:

Search #1: (((((chronic disease) OR chronic disease[MeSH Terms]) OR noncommunicable disease) OR non-communicable disease) OR noncommunicable disease[MeSH Terms]) AND Indonesia

Search #2: ((cancer) OR cancer[MeSH Terms]) AND Indonesia

Search #3: ((diabet*) OR diabetes mellitus[MeSH Terms]) AND Indonesia

Search #4: ((((((cardiovascular disease) OR cardiovascular diseases[MeSH Terms]) OR stroke) OR stroke[MeSH Terms]) OR myocardial infarction) OR myocardial infarct[MeSH Terms]) AND Indonesia

Search #5: ((((((chronic obstructive pulmonary disease) OR chronic obstructive pulmonary disease[MeSH Terms]) OR copd[MeSH Terms]) OR copd) OR lung disease) OR lung disease[MeSH Terms]) AND Indonesia

Search #6: ((((high blood pressure) OR high blood pressure[MeSH Terms]) OR hyperten*) OR hypertension[MeSH Terms]) AND Indonesia

Search #7: ((((overweight) OR overweight[MeSH Terms]) OR obes*) OR obesity[MeSH Terms]) AND Indonesia

Search #8: ((dyslipidemia) OR dyslipidemia[MeSH Terms]) AND Indonesia

Search #9: ((cholesterol) OR cholesterol[MeSH Terms]) AND Indonesia

Search #10: (((((smoking) OR smoking[MeSH Terms]) OR smok*) OR tobacco) OR tobacco[MeSH Terms]) AND Indonesia

Search #11: ((alcohol*) OR alcohol drinking[MeSH Terms]) AND Indonesia

Search #12: ((((exercise) OR physical exercise[MeSH Terms]) OR physical activ*) OR physical activity[MeSH Terms]) AND Indonesia

Search #13: ((((food habit) OR food habit[MeSH Terms]) OR unhealthy diet) OR eating behaviors[MeSH Terms]) AND Indonesia

Search #14: (((disability) OR disabled) OR disabilities, people with[MeSH Terms]) AND Indonesia

Search #15: ((((((activities of daily living) OR *ADL) OR activities of daily living[MeSH Terms]) OR instrumental activities of daily living[MeSH Terms]) OR instrumental activities of daily living) OR IADL) AND Indonesia

Search #16: ((functional health) OR functional health[MeSH Terms]) AND Indonesia

Search #17: ((((((multimorbidity) OR multimorbidity[MeSH Terms]) OR multimorb*) OR comorbid*) OR comorbidities[MeSH Terms]) OR comorbidity[MeSH Terms]) AND Indonesia

Filters: ("2000/01/01"[Date - Publication] : "2015/10/01"[Date - Publication]) AND

(English[Language]) OR Indonesian[Language]

Combined searches #1- #17: 3049 search results (last run October 15, 2015):

((((((((((((((((((((((chronic disease) OR chronic disease[MeSH Terms]) OR noncommunicable disease) OR non-communicable disease) OR noncommunicable disease[MeSH Terms]) AND Indonesia)) OR (((cancer) OR cancer[MeSH Terms]) AND Indonesia)) OR (((diabet*) OR diabetes mellitus[MeSH Terms]) AND Indonesia)) OR (((((((cardiovascular disease) OR cardiovascular diseases[MeSH Terms]) OR stroke) OR stroke[MeSH Terms]) OR myocardial infarction) OR myocardial infarct[MeSH Terms]) AND Indonesia)) OR (((((((chronic obstructive pulmonary disease) OR chronic obstructive pulmonary disease[MeSH Terms]) OR copd[MeSH Terms]) OR copd) OR lung disease) OR lung disease[MeSH Terms]) AND Indonesia)) OR (((((high blood pressure) OR high blood pressure[MeSH Terms]) OR hyperten*) OR hypertension[MeSH Terms]) AND Indonesia)) OR (((((overweight) OR overweight[MeSH Terms]) OR obes*) OR obesity[MeSH Terms]) AND Indonesia)) OR (((dyslipidemia) OR dyslipidemia[MeSH Terms]) AND Indonesia)) OR (((cholesterol) OR cholesterol[MeSH Terms]) AND Indonesia)) OR ((((((smoking) OR smoking[MeSH Terms]) OR smok*) OR tobacco) OR tobacco[MeSH Terms]) AND Indonesia)) OR (((alcohol*) OR alcohol drinking[MeSH Terms]) AND Indonesia)) OR (((((exercise) OR physical exercise[MeSH Terms]) OR physical activ*) OR physical activity[MeSH Terms]) AND Indonesia)) OR (((((food habit) OR food habit[MeSH Terms]) OR unhealthy diet) OR eating behaviors[MeSH Terms]) AND Indonesia)) OR ((((disability) OR disabled) OR disabilities, people with[MeSH Terms]) AND Indonesia)) OR (((((((activities of daily living) OR *ADL) OR activities of daily living[MeSH Terms]) OR instrumental activities of daily living[MeSH Terms]) OR instrumental activities of daily living) OR IADL) AND Indonesia)) OR (((functional health) OR functional health[MeSH Terms]) AND Indonesia)) OR (((((((multimorbidity) OR multimorbidity[MeSH Terms]) OR multimorb*) OR comorbid*) OR comorbidities[MeSH Terms]) OR comorbidity[MeSH Terms]) AND Indonesia)

---
